# A qualitative study of cardiac rehabilitation patients’ perspectives on taking medicines: implications for the ‘medicines-resistance’ model of medicine-taking

**DOI:** 10.1186/1472-6963-13-302

**Published:** 2013-08-09

**Authors:** Simon White, Paul Bissell, Claire Anderson

**Affiliations:** 1 School of Pharmacy, Keele University, Staffordshire ST5 5BG, UK; 2 School of Health and Related Research, University of Sheffield, 30 Regent Street, Sheffield S1 4DA, UK; 3 School of Pharmacy, University of Nottingham, Nottingham NG7 2RD, UK

## Abstract

**Background:**

The appropriate use of medicines continues to be an important area of inter-disciplinary research activity both in the UK and beyond. Key qualitative work in this area in the last decade has included the ‘medicines resistance’ model of medicine-taking, which was based on a meta-ethnography of 37 qualitative studies. This model proposed that patients approach medicine-taking as ‘passive accepters’, ‘active accepters’, ‘active modifiers’ or ‘complete rejecters’, of which the latter two categories were considered to show ‘resistance’ to medicines. However, critical assessment of the model appears to be currently lacking, particularly in terms of its use in clinical practice. This paper seeks to contribute to the literature in this area by critically examining the practical application of the model in light of the findings from a qualitative, follow-up study of cardiac rehabilitation patients’ perspectives and experiences of using medicines.

**Methods:**

Following ethical approval, in-depth, audiotaped, qualitative interviews were conducted with fifteen patients who had completed a UK hospital-based cardiac rehabilitation programme. Participants were aged 42–65, white British and from a variety of socioeconomic backgrounds. Interview topics included perspectives on coronary heart disease, medicine-taking and lifestyle changes. Follow-up interviews with ten patients approximately nine months later explored whether their perspectives had changed.

**Results:**

The findings suggest that the active/passive and accepter/modifier distinctions may not allow for clear determination of which profile a patient fits into at any given point, and that definitions such as ‘accepter’ and ‘resistance’ may be insufficiently discerning to categorise patients’ use of medicines in practice. These problems appear to arise when the issue of patients’ accounts about medicines adherence are considered, since patients may have concerns or disquiet about medicines whether or not they are adherent and the model does not consider disquiet in isolation from adherence.

**Conclusions:**

Practical application of the ‘medicines resistance’ model of medicine-taking may be problematic in this patient group. Dissociation of disquiet about medicines from medicines adherence may allow for a focus on helping patients to resolve their disquiet, if possible, without this necessarily having to be viewed in terms of its potential effect on adherence.

## Background

Lay peoples’ use of the medicines they are prescribed (or purchase) remains an area of study that continues to generate a huge empirical literature. Numerous studies have demonstrated that people find following medication regimens problematic, that various lifestyle, psychological and social factors are implicated in this, that inappropriate medication use adversely impacts on patients’ health outcomes and hospital admissions, and the costs of this to health care systems are highly significant [1]. Various interventions and policies have been proposed to address this, but few simple and effective solutions have been identified [2]. However, in the previous twenty years there has been an increasing emphasis in the empirical and policy field on firstly challenging the terms and assumptions underpinning what has been referred to as ‘compliance’ research and secondly, exploring how relationships between prescriber and medicines user might be re-configured to ensure appropriate use of medicines [3,4]. For example, there have been calls to move away from what has been regarded as the rather rigid and professionally focused term compliance to embrace adherence and the notion of concordance has emerged as a way of illustrating the importance of understanding the users’ perspectives in relation to medication practices [3,5]. Despite this, the basic problem of how people orientate themselves to their medicines and their medication regimens remains.

**Figure 1:**
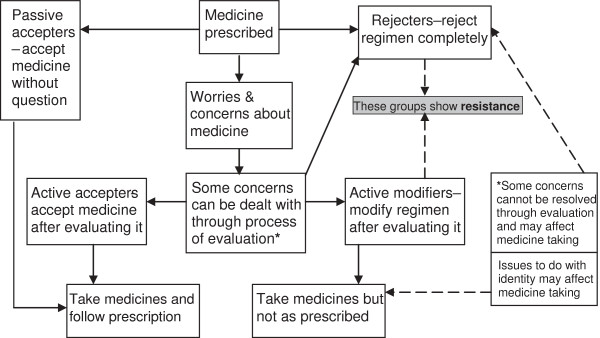
**Pound et al’s ‘medicines resistance’ model of medicine-taking [**[6].

To understand this area in more depth, researchers have increasingly been drawn to qualitative approaches and one of the most interesting papers to have emerged in this field in recent years is that by Pound et al [6]. Their ‘medicines-resistance’ model of medicine-taking (Figure 1) was developed from a meta-ethnography [7] of 37 qualitative research studies published between 1992 and 2001 that primarily explored lay peoples’ views of medicine-taking. Their model placed peoples’ approaches to prescribed medicines into the four categories of ‘passive accepter’ (accepts medicines without question), ‘active accepter’ (accepts medicines after self-evaluation), ‘active modifier’ (modifies their medicines regimen after self-evaluation) and ‘complete rejecter’ (rejects taking medicines completely). Of these categories, the latter two were considered to show what they described as ‘medicines-resistance’. The ‘medicines-resistance’ model differed from Dowell and Hudson’s model of medicine-taking in that it included the category of ‘active accepter’ of medicines [8]. This is an important distinction because although Dowell and Hudson found that most patients evaluated medicines for themselves before accepting them, their model only categorised such acceptance as a passive process [8]. In contrast, the ‘medicines-resistance’ model recognised this as an active process, as is modification of the medication regimen after a process of self-evaluation.

This paper aims to critically examine the practical application of the ‘medicines resistance’ model of medicine-taking in light of the findings of a qualitative, follow-up study of cardiac rehabilitation (CR) patients’ perspectives and experiences with medicines-taking. Critical assessment of the model appears to be currently lacking and CR patients are a contrasting patient group from those included in Pound et al’s meta-ethnography [6]. CR is a multidisciplinary coronary heart disease (CHD) secondary prevention strategy principally involving structured exercise, psychosocial support and information provision (on various topics including medicines) that has been shown to have health benefits for patients who have had a cardiac event (e.g. myocardial infarction) or surgical intervention [9]. Studies have not assessed whether CR patients’ perspectives align with the ‘medicines-resistance’ model and no clear picture is apparent in the available research on this point; some studies report that CR patients appear to be adherent with medicines, at least up to six months after CR programme attendance [10], whereas other studies suggest that adherence declines in the first three years, although perhaps less or more slowly compared to patients who did not attend CR [11]. With these issues in mind, the discussion now turns to the approach and the methods used in the present study.

## Methods

A qualitative approach to the study was taken on the grounds of being better suited to exploring the range, depth and complexity of people’s perspectives than a quantitative approach [12]. In-depth interviews were used because this technique enables individual participants’ perspectives to be explored in detail [13]. Lincolnshire NHS Research Ethics Committee approval for the study was obtained.

A purposive sample of patients were recruited from a UK district general hospital-based CR programme, which ran for six weeks on a one session per week basis. The programme involved multidisciplinary input from health professionals such as nurses, physiotherapists, occupational therapists, dietitians, pharmacists and social workers. This included assessment of individual physical, psychological and social needs. It also involved provision of structured exercise sessions and information on various topics including lifestyle modification recommendations (e.g. smoking cessation and adopting a healthy diet and exercise regime), stress management, local CHD patient groups, and the use, benefit and harms of medicines [14]. Each session included group-based information provision on one of these topics for approximately 45 minutes, followed by structured exercises and relaxation.

All patients who attended the CR programme were considered eligible to participate, provided that written consent was given. The first author attended the CR programme to explain the study to patients and distribute Information Sheets. On successive weeks the first author repeated this process, but also waited at the end to give patients an opportunity to volunteer. Written consent and a contact telephone number were obtained. Each patient was then contacted to arrange an interview location of their choice and a mutually convenient time. The initial interviews were conducted after patients had finished attending the CR programme, which was approximately three months after hospital discharge. Fifteen patients were interviewed. This number was determined by the point of apparent saturation of the data [15].

The interview guide used was based on a review of the literature. The topics included patients’ perspectives on CHD and the CR programme, and their perspectives on lifestyle modification and medicine-taking. Open, non-leading questions about these topics were asked. These questions were intended only to serve as prompts for the topics of interest; other questions were often asked that were not specifically in the guide or were asked in a slightly different way to the guide. This was so that patients could talk about their experiences and perspectives as they wished and express themselves in their own words with minimal prompting. Questions were asked at points that flowed from what patients said, generally to clarify or explore the responses given, rather than sticking rigidly to the order in the guide. This ensured that the topics of interest were covered, but allowed issues of importance to interviewees to emerge. The first author conducted the interviews and made detailed field notes after each interview of significant events and non-verbal cues to aid interpretation of the data. The interviews were audiotaped and transcribed verbatim.

Re-recruitment of the patients for follow-up interviews started approximately nine months after the initial interview. Hospital records were checked to determine whether any of the patients had died. Fourteen patients were subsequently contacted in writing to invite them to participate, of which ten were recruited. The same interview topics were used, although the focus was on whether patients’ views had changed since the initial interview. All interviews were audiotaped and transcribed verbatim.

Data analysis began by reading and repeated re-reading of the transcripts to identify similarities. Drawing on the principles of grounded theory, concepts embodied in these similarities were grouped into themes and scrutinised to identify the properties or characteristics of each theme [15]. Further interview transcripts were compared with the themes identified to examine the similarities and differences in detail [15]. Themes were modified accordingly so that the properties of each were more clearly defined or refined [15,16]. Themes were compared with each other and relationships between them identified and examined in detail in order to develop an overall structure [15,16]. Care was taken to account for views or experiences that differed from the majority view, which resulted in the properties of themes being further refined and a clearer interpretation of the data [13,16]. The first author kept a journal of reflections and thoughts about each interview and interpretations of the data, which included diagrams of possible relationships between emerging categories to guide or reflect the analysis [13]. The themes were further refined through discussion between the authors. A considerable amount of time was spent thinking about the relationships between concepts in the data, the properties of themes and variations in the data. This in turn prompted further scrutiny of the transcripts and further examination of the themes to ensure that the analysis was thoroughly grounded in the data. This process continued throughout the study up to and including the stages of writing up, as further insights were gained [16].

## Results

Eleven of the participants were men and four were women. All were White British within the age range of 42–65 and from a variety of socioeconomic backgrounds. Thirteen were married and living with a partner, whilst two patients lived alone. All of the patients had attended the CR programme because they had had a heart attack (the term heart attack is used here in preference to myocardial infarction because this was the term that patients used). Patients’ descriptions of their other medical conditions were used, as specific medical diagnoses were not usually known. Details of patients’ medicines were compiled from patients’ memory and, where available, the medicines themselves, repeat prescriptions and patients’ own medication lists. These usually included aspirin, a statin, a beta-blocker, an ACE inhibitor, and glyceryl trinitrate spray. Seven patients were taking alternatives to these medicines or additional medicines for chest pain (e.g. nitrates).

All of the patients were keen to report that they were taking their medicines as prescribed, although we recognise that it cannot be said that this was definitely so, given that these were self-reports. Having said this, patients demonstrated that they were taking their medicines in one or more of four ways: by specifically pointing out that they were prepared to take them; by talking about having a strategy to remember to take them; by showing that where doses had not been taken as usual, this had not been intentional; and by tolerating or seeking medical advice about side effects, rather than deciding not to continue taking the medicines thought to be responsible.

As such, none of the patients were clearly ‘rejecters’ or ‘active modifiers’ of their medicines as described in the medicines resistance model, so the discussion now considers whether their acceptance of medicines aligns with the ‘medicines-resistance’ model, starting with ‘passive acceptors’. We draw on the medicines resistance typology to guide our analysis.

### ‘Passive acceptance’ of medicines?

In the initial interviews, six patients appeared to accept their medicines or at least did not express any particular concerns about taking them, and neither did they talk about wanting more information about their medicines than they had been given. There were no obvious differences between their medicines and those of the rest of the patients (e.g. they were not taking fewer medicines than the others). The situation was much the same in the follow-up series of interviews, as five of the original six patients still did not express any worries or concerns about taking their medicines (the sixth patient did not participate in the follow-up interviews). However, the extent to which this could be considered a passive process was open to question, since patients appeared to have undertaken some form of evaluation and, in the absence of any problems that concerned them, appeared to have accepted the need to take their medicines. This was exemplified by one patient who said:

*“I couldn’t tell you what this one actually does and what that one does, although at the time I read what this one did and what that one did…I’ve a good idea that as long as you’re feeling alright they’re doing whatever it is they’re supposed to do and do you really need to know what they’re doing?”* (R10: male)

Another patient pointed out about his medicines that the doctor had not “given them to me for nothing” and ‘so I’ll put them down my neck as long as they say I’ve got to do it’. As a further example, a patient talked about being given a booklet about her medicines and so knew that ‘this one’s for your heart, that one’s for diabetes and that’. Subsequently, she pointed out that:

*“…quite obviously what I’ve been prescribed is supposed to be helping me, so therefore it’s up to me to keep taking them isn’t it; keep taking the tablets.”* (R3: female)

On the other hand, there also appeared to be a strong sense that these patients felt they ought to trust and accept their doctors’ prescribing decisions and instructions, in other words, in line with a Parsonian view of the doctor – patient relationship. Within this group of patients, there was a clear desire to present themselves as following the doctors’ instructions and demonstrate ‘compliance’. For example, one patient pointed out that “if he [doctor] tells me I’ve got to take [medicines] that’s all I need to know, not what specific purpose it’s doing”. As far as he was concerned “the people who should know about that are the doctors, they should know the milligrams and things like that”.

Nevertheless, applying the passive/active acceptance distinction in the ‘medicines-resistance’ model to these patients seemed problematic, as their self-evaluation of medicines suggested an almost continuously active process of evaluation and monitoring, but in other respects their approach could be seen as being passive in that they reporting taking their medicines as prescribed. However, the other patients expressed more significant concerns about taking medicines, and the discussion now focuses on whether their approach to these concerns aligns with the medicines resistance model.

### ‘Active acceptance’ of medicines?

In the initial interviews, nine patients (i.e. over half) spontaneously expressed worries and concerns about taking medicines and we have grouped these into four areas: side effects; how long they would need to take the medicines for; differences in information from written sources of information compared to that provided by health professionals; and, why doses of certain medicines frequently changed. The issues in applying the ‘medicines-resistance’ model here concerned the extent to which patients could be considered to have accepted their medicines in the absence of resolution of their concerns (i.e. whether they did accept them), and more significantly that some patients sought medical advice because of difficulty in tolerating side effects (i.e. whether they modified their regimen). The following examples illustrate these points.

Three patients raised concerns as a result of experiencing side effects. Of these, one patient in particular said that he had had “problems with tablets”. He had subsequently taken a keen interest in the potential side effects of his medicines and said the information he had been given had not given enough indication of the likelihood of getting any of the side effects listed. This had been compounded by the sheer number of potential side effects. The underlying significance was that the information had not helped him determine whether or not the “discomfort” he had been experiencing in his chest was a side effect of the alternative medicine (losartan) he had been prescribed when an initial choice had given him a dry tickly cough, whereas he had been able to “quickly pick up on” this cough being a side effect of the initial choice (ramipril). He had sought medical advice because he was unable to tolerate the cough. The situation became more confusing when he was later told (by another patient) that the alternative “doesn’t work as well” as the initial choice because this made him wonder whether this was why he was getting “discomfort”, rather than it being a side effect. Nevertheless, he pointed out that he was continuing to take his medicines as prescribed.

The second patient talked about wanting to know the likelihood of getting side effects from her medicines because she too had been having difficulty in tolerating the dry tickly cough induced by the same initial choice of medicine (ramipril), and after seeking medical advice was due to start taking an alternative. Her concern seemed to be that she might also experience side effects from the alternative choice. The third patient said that she found that when she took one of her medicines (atenolol) it made her “feel a bit queer sometimes” and described experiencing symptoms suggestive of postural hypotension. This seemed to have been what had caused her to take more interest in the atenolol than her other medicines but she said that the information she had been given had not helped her to understand why it had been prescribed.

Three patients talked about wanting to know how long they would have to take their medicines for, which suggested concern about the potential to be taking medicines indefinitely, although there was no suggestion that this had affected their decision to continue taking them. For example, one patient said “It’s waiting just how long I’ve got to take them, is it going to be forever and a day?”, whilst another said that he had wanted to know:

*“…for how long you’re going to have to take them. I accept that most of them I’ll be taking for the rest of my life…but I was told maybe six to twelve months, this is the basic time for drugs. So we’ll have to see what happens after that.”* (R13: male)

Two patients talked about being concerned about differences in the information between written sources of information or differences of opinion between health professionals about their medicines. One said that he had read a book about CHD that his GP had recommended and said that this had caused “an element of uncertainty” over why certain medicines-related aspects differed from his own circumstances. He said that he had asked his GP about these but it seemed that this had not fully answered all of his questions, such as that “it says nobody should really be on above seventy-five [milligrams] of aspirin, well I’m on double that”. He knew that this was because it was what his consultant “recommends”, but this still did not explain why his consultant’s view differed from what he had read in the book. He also said “it worried me taking eight different types of tablets” because “it does say in the book that the most tablets patients would normally be on is three or four tablets a day”. This seemed to have worried him because it did not appear to be consistent with having had a “mild heart attack”, as he said he had been told when he had been in hospital. Nevertheless, he pointed out that he was continuing to take these medicines. The other patient said that she was “not quite sure” why there seemed to be a difference of opinion between her GP and her consultant about whether she should be on a beta-blocker or an ACE inhibitor and said that although she was continuing to take the beta-blocker, at her next “check up” with her consultant she intended to ask about whether she should be prescribed an ACE inhibitor.

One patient said that what he had particularly wanted to know was why his dose of warfarin had been increased when he last went to have his “blood checked”, since this had not been explained to him at the time. He said that he wanted information about “why you’ve got to reduce it, why you’ve got to increase it” so that he could understand why:

*“…it’s getting thinner or if it’s getting thicker, if there’s anything they can tell you, maybe I’m doing something wrong…maybe there’s something I can do myself to help my blood stay at that sort of level…”* (R1: male)

It seemed that the main reason why he wanted to know this was because he had been worried that his blood not being “at that sort of level” meant that there was something wrong that might lead to another heart attack. He reported that he had been taking this higher dose, and still hoped that he would be given an explanation at some point.

In the follow-up interviews the same issues in applying the ‘medicines-resistance’ model were encountered (i.e. whether patients accepted their medicines despite concerns not being resolved, and whether they modified their regimen). The patients who were concerned about side effects in the initial interview continued to have concerns, but all of the patients said that they were continuing to take their medicines as prescribed. For example, one of these patients explained that:

*“When I changed the tablets over from ramipril to losartan, oh it took me a long time to get used to them and I didn’t feel as well on them as I did on ramipril. I couldn’t really explain it, not very well in myself. It was a question of energy…[ramipril] is tried and true and I think it works and I don’t know about losartan whether it does or not. I don’t cough as much but was it as effective as the ramipril?”* (R2: female)

Her description of this dilemma suggested that she had not really accepted the alternative choice of medicine, but in continuing to take it as prescribed, she was not ‘resistant’ to it either.

The patient who was concerned about getting “discomfort” in his chest had eventually been told that the discomfort was “all to do with indigestion” rather than being related to his heart. He explained that subsequently other “tablets started that way” and he had not experienced the “discomfort” since. This seemed to have caused him to question whether certain heart-related medicines that had been started or had doses increased to deal with this “discomfort” were necessary because they had not relieved his “discomfort”. He said that he had “badgered” his GP to reduce these medicines because he did “want to alter them but I only want to do it through my GP so he knows what’s happening”, rather than just deciding not to take them. The principal difficulty in applying the ‘medicines-resistance’ model here was that on the one hand he accepted and took his medicines as prescribed, but on the other hand he had a clear interest in his medicines regimen being modified to avoid side effects. However, he was not modifying (and hence was not showing ‘resistance’ to) his medicines *per se*, neither was he modifying his regimen without his doctor’s knowledge. The issues raised by these findings and the implications for the medicines-resistance model are now discussed in more detail.

## Discussion

These findings suggest that, since all the of patients reported taking their medicines as prescribed, it is difficult to place them into the category of either ‘complete rejecter’ or ‘active modifier’, and in practically applying the ‘medicines-resistance’ model the distinctions of passive/active and accepter/modifier became problematic, as did the definitions of ‘accepter’ and ‘resistance’. It is possible that these issues are more apparent in the context of CHD patients, since no papers concerning CHD were included in Pound et al’s meta-ethnography [6], which may suggest greater disease specificity than the model currently allows for. We accept that the results of this study are based on the participants’ expressed views and so may not necessarily have wider applicability and that there is the possibility of a social desirability effect, where the patients responded in a way dependent on who they perceived the interviewer to be. However, it is also possible that the ‘medicines-resistance’ model of medicine-taking, as it currently stands, does not adequately account for the complexity of medicine-taking behaviour as characterised through the experiences of CR patients.

The problem with determining whether some patients had ‘passively accepted’ their medicines was that although they had apparently deferred to their doctor’s judgement, they were also found to have undergone a process of almost continuous self-evaluation of their medicines. This was so, even if only in terms of checking that they were ‘feeling alright’. Since this is suggestive of an active process, categorising them as having accepted their medicines ‘without question’ becomes problematic. Furthermore, choosing to defer to the doctor’s judgement is arguably more an active process of acceptance than passive. The issue with the accepter/modifier distinction was that some patients sought medical advice because of intolerable side effects but continued to take the medicines thought to be responsible unless the doctor prescribed an alternative. It remains unclear as to whether ‘active acceptance’ can reasonably include seeking medical intervention with the hope of getting a particular medicine stopped or changed, but since they were prepared to take alternatives, categorising them as ‘resistant’ is also problematic. However, it could be argued that this is more suggestive of ‘active modification’ than ‘active acceptance’, but if so it would perhaps be better categorised as ‘modification by proxy’, since the decision to modify was made by the doctor after a problem had been identified by the patient. The point made in the ‘medicines-resistance’ model (Figure 1) that ‘some concerns cannot be resolved through evaluation and may affect medicine-taking’ does not clarify these issues, as the model only appears to show this leading to rejection of medicines. Britten argues that the profiles of ‘accepter’, ‘modifier’ and ‘rejecter’ do not have to be considered as being static, since people may change their approach to medicines [17], but this study highlights the more fundamental problem in determining which category a patient may fit at any given point.

Determining whether the ‘medicines-resistance’ model could be applied to the patients in this study was also hindered by the lack of clear definitions of what might constitute ‘resistance’ and what constitutes an ‘accepter’. The model (Figure 1) indicates that ‘resistance’ is shown by those who completely reject or modify their prescribed medicines regimen, whereas Pound et al. later referred to ‘resistance’ in the rather broader context of a ‘considerable reluctance to take medicine and a preference to take as little as possible’ [6]. The findings of this study indicated that over half of the patients expressed worries and concerns about taking medicines, which in this broader context could perhaps be viewed as ‘resistance’ even though all patients reported taking their medicines as prescribed (although we accept that there may be differences between their accounts and actual practice). The problem with the notion of ‘acceptance’ concerns the extent to which patients could be considered to have accepted their medicines in the absence of resolution of their worries and concerns, despite self-evaluation. Morgan considered this to be ‘problematic adherence’ in her study of hypertensive patients (included in Pound et al’s meta-ethnography) and explained this by saying that although patients took their medicines, they were not ‘reconciled’ to using them long-term, worried about the potential for long-term adverse effects and disliked ‘feeling addicted’ to them [18]. This implies that patients had not *accepted* their medicines any more than patients had in the present study, unless a very broad understanding of the term acceptance is employed.

Despite these apparent limitations of the ‘medicines-resistance’ model, the findings from the study presented here do support Pound et al’s work [6], insofar as that most patients expressed concerns about taking medicines, especially concerning the potential for them to cause harm. Subsequently, patients tended to take a particular interest in medicines information about these issues. This has been reported by other studies of CR patients, and CHD patients who may not have attended a CR programme [19-21]. It has also been reported in the literature more generally [22]. What perhaps many patients in this study were showing was a sense of discontent, or disquiet, about their medicines, for a variety of reasons but particularly about side effects. This was coupled with a keen desire to avoid further CHD-related events, awareness of the subsequent need to take regular medicines and a strong sense of acceptance towards their doctor’s prescribing expertise. In some instances, as has been discussed, their disquiet prompted patients to seek medical advice with the hope of the medicine being stopped or an alternative prescribed. This perhaps goes beyond Morgan’s concept of ‘problematic adherence’ [18], although it should be pointed out that patients reported continuing to take the medicines thought to be causing their disquiet, unless prescribed an alternative.

The problems inherent in practically applying the ‘medicines-resistance’ model appear to arise when adherence is taken into account, since features of ‘resistance’ to medicines (i.e. what we term disquiet) may be seen in patients who because they appear to be adherent would not otherwise be categorised as ‘resistant’, as has been shown in this study. Norreslet et al described medicines being viewed as a ‘necessary evil’ amongst patients with atopic dermatitis, who appeared to have gone through periods of what could be viewed as ‘resistance’ to certain medicines (e.g. topical corticosteroids) because of their side effects [23]. Nevertheless, patients eventually felt that they had no choice but to use these medicines because there were no other effective treatments. The point here is not that they had changed from being ‘resistant’ to being ‘active accepters’, in line with Britten’s point about the ‘medicines resistance’ model profiles not being static [17], but that their considerable disquiet continued, and that this was a central feature of their accounts of using medicines. In contrast, the CR patients in the present study did have effective alternatives available, which reduced the ‘evil’, whilst preserving the ‘necessary’. McCoy has similarly written about the work involved for patients in ‘doing adherence’ to antiretroviral medicines for HIV and described features that could be considered as ‘resistance’ [24]. Here again these features may be better understood as disquiet, since patients were to all intents and purpose adherent, whilst doing the work of successfully accommodating medicine-taking into their everyday lives. In terms of the implications for practice, this suggests that a more fruitful approach would be to dissociate disquiet about medicines from non-adherence to medicines and where patients do require support with adherence, the UK National Institute of Health and Clinical Excellence guidance, amongst other resources, offers a practical approach [25]. When viewed in this way the notion of ‘medicines-resistance’ may become less relevant and the focus can shift onto helping patients to resolve their disquiet, where possible, without this necessarily being viewed in terms of the potential effect on non-adherence.

## Conclusions

In this paper we have argued that the practical application of the ‘medicines resistance’ model of medicine-taking appears to be problematic for several reasons. The active/passive and accepter/modifier distinctions may not allow for clear determination of which profile a patient may fit at any given point, and that the definitions of ‘accepter’ and ‘resistance’ may be insufficiently discerning to categorise patients’ use of medicines in practice. We have pointed out that these problems arise when the issue of adherence is taken into account, since the ‘medicines resistance’ model does not consider disquiet about medicines in isolation. Subsequently, since disquiet may well be a feature of both adherence and non-adherence to medicines, dissociation of disquiet from adherence may allow for a focus on helping patients to resolve their disquiet, if possible, without this necessarily having to be viewed in terms of its potential effect on adherence. As such, we suggest that a more fruitful approach may be to focus more on patients’ disquiet about medicines and less on whether or not they are ‘resistant’.

## Competing interests

The authors declare that they have no competing interests.

## Authors’ contributions

All authors participated in the conception and design of the study. SW conducted all of the interviews. All authors participated in analysis of the data and drafting of the manuscript. All authors read and approved the final manuscript. This was SW’s PhD study and as such was funded by SW and the University of Nottingham. The views expressed in this paper are those of the authors.

## Pre-publication history

The pre-publication history for this paper can be accessed here:

http://www.biomedcentral.com/1472-6963/13/302/prepub
